# Re: “Late Presentation of a 9mm Bullet in the Ureteropelvic Junction Causing Acute Renal Failure in a Solitary Functioning Left Kidney” by Jhaveri and D'Angelo (*J Endourol Case Rep.* 2018;4:173–175)

**DOI:** 10.1089/cren.2018.0114

**Published:** 2019-03-18

**Authors:** Ines M. Pina, Ahmed M. Omar, Michael S. Floyd

**Affiliations:** Department of Reconstructive Urology, St. Helens and Knowsley Hospital NHS Trust, Whiston Hospital, Liverpool, United Kingdom.

**Dear Editor,**

We read with interest the recent article by Jhaveri and D'Angelo.^1^ A case of a 53-year-old man with a solitary kidney is reported after previous gunshot injuries who presented with left flank pain and renal failure. Subsequent imaging revealed a hydronephrotic left kidney with a 1.5 cm calcification in the left pelviureteric junction (PUJ), which was deemed to be a stone encasing a bullet and an atrophic kidney. The operative management comprising stent insertion, delayed definitive treatment necessitating holmium laser tripsy, and eventual extraction of the bullet fragment with graspers is described.^1^ The case is notable as the clinical sequelae of delayed gunshot injury are illustrated as 10 years elapsed between injury and eventual presentation because of delayed migration of the bullet fragment into the PUJ. The authors do not state whether any follow-up imaging was undertaken or whether delayed ureteral stricturing occurred.

The rarity of such cases is alluded to by the authors and they further acknowledge the difficulty in diagnosing late urologic complications of gunshot injury.

The authors should recognize the heterogeneity of presentations that exist with gunshot injuries and the upper urinary tract. Spontaneous passage per urethra of a shell fragment 23 years after initial injury has been described^2^ and Gawande et al. have also described a case of spontaneous passage of a gunshot pellet 2 hours after injury.^3^ The passage of multiple spontaneous pellets has also been described.^4^

A 5-month interval between initial injury necessitating open surgical repair and delayed presentation to a urologic service with colic has been described with one case passing the pellet spontaneously and one case requiring nephrostomy and eventual ureteroscopic extraction with a grasper.^5,6^

Gutman and colleagues have reported a case of delayed colic caused by gunshot occurring 7 years after injury.^7^ Effective use of the holmium laser through a percutaneous approach to shatter a bullet fragment thus permitting basket extraction^8^ has also been described with a 4-month stricture-free follow-up. Finally, all urologists who encounter delayed gunshot injuries should be aware of the fact that bullets may not follow anatomical routes as described by Kollias and Kyriakopoulos^9^; although the entry point was the supraclavicular area, the bullet was eventually removed from the urethra!
